# P3HB from CH_4_ using methanotrophs: aspects of bioreactor, fermentation process and modelling for cost-effective biopolymer production

**DOI:** 10.3389/fbioe.2023.1137749

**Published:** 2023-06-19

**Authors:** Parya Safaeian, Fatemeh Yazdian, Kianoush Khosravi-Darani, Hamid Rashedi, Maximilian Lackner

**Affiliations:** ^1^ Department of Life Science Engineering, Faculty of New Sciences and Technologies, University of Tehran, Tehran, Iran; ^2^ Department of Food Technology Research, National Nutrition and Food Technology Research Institute, Shahid Beheshti University of Medical Sciences, Tehran, Iran; ^3^ School of Chemical Engineering, College of Engineering, University of Tehran, Tehran, Iran; ^4^ Circe Biotechnologie GmbH, Vienna, Austria

**Keywords:** bioreactor, poly-3-hydroxybutyrate (P3HB), polyhydroxyalkanoates (PHA), vertical tubular loop bioreactor, horizontal tubular loop bioreactor (HTLB), forced liquid flow

## Abstract

P3HB (poly-β-hydroxybutyrate), an energy-storage compound of several microorganisms, can be used as bioplastics material. P3HB is completely biodegradable under aerobic and aerobic conditions, also in the marine environment. The intracellular agglomeration of P3HB was examined employing a methanotrophic consortium. Supplanting fossil, non-degradable polymers by P3HB can significantly reduce the environmental impact of plastics. Utilizing inexpensive carbon sources like CH_4_ (natural gas, biogas) is a fundamental methodology to make P3HB production less costly, and to avoid the use of primary agricultural products such as sugar or starch. Biomass growth in polyhydroxyalkanoates (PHA) in general and in Poly (3-hydroxybutyrate) manufacture in specific could be a foremost point, so here the authors focus on natural gas as a proper carbon source and on the selection of bioreactors to produceP3HB, and in future further PHA, from that substrate. CH_4_ can also be obtained from biomass, e.g., biogas, syngas methanation or power-to-gas (synthetic natural gas, SNG). Simulation software can be utilized for examination, optimizing and scale-up of the process as shown in this paper. The fermentation systems continuously stirred tank reactor (CSTR), forced-liquid vertical loop bioreactor (VTLB), forced-liquid horizontal tubular loop bioreactor (HTLB), airlift (AL) fermenter and bubble column (BC) fermenter were compared for their methane conversion, kLa value, productivity, advantages and disadvantages. Methane is compared to methanol and other feedstocks. It was discovered that under optimum processing circumstances and using *Methylocystis hirsuta*, the cells accumulated 51.6% cell dry mass of P3HB in the VTLB setup.

## 1 Introduction

In recent years, sustainability issues related to plastics production, consumption and end-of-life have taken center stage. Products made from conventional plastics release microplastics and nanoplastics during their use phase (e.g., tires on roads, garments upon washing) and when starting to fragment after being leaked into the environment. On a global scale, only 9% of all plastics today are recycled—and double the amount is leaked. It is not only the mismanagement of plastics, but their intrinsic properties of being durable which cause the microplastics crisis; Microplastics can carry pathogens, heavy metals, adsorbed organic toxins—and additives contained in the formulations—into the human body. Exposure routes are via inhalation and ingestion. What’s more, fossil-derived plastics contribute to climate change through their energy-intense manufacturing and by waste incineration, a common end-of-life scenario. The positive aspects of plastics in daily life are not overlooked, but they must not make us blind about their detrimental effects on virtually all ecosystems.

Today, almost every industry sector uses plastic materials on a regular basis in a multitude of applications. The business model is almost exclusively linear, because plastics are very cheap to procure and to process—and environmental and social costs are externalized.

Large amounts of plastics are improperly handled and released into the environment, mostly in packaging applications and other short-lived products. On a global level, the very low rate of plastics recycling could not be raised in the last three decades, and it is mainly limited to in-process recycling and hardly includes lost consumer waste, with single-use PET bottles being an exception.

Bioplastics can offer a solution. The study of bio-based and biodegradable polymers is expanding quickly, and bioplastics have reached a market share of approx. 2% (Shimao, 2001). Hence bioplastics still constitute a niche market today. Due to their long half-lives in nature and the fact that a sizable portion is left in nature rather than being recycled, (fossil and non-degradable) traditional plastics have been realized as highly problematic, with health impacts being still hardly understood, and their recycling alone is not sufficient, even if it was applied tenfold to today’s situation. Manufacturing of non-biodegradable polymers, such as polystyrene (PS), polyethyleneterephthalate (PET), polyethylene (PE), and polypropylene (PP), begins with fossil-based monomers (Moradi et al., 2019). In comparison to non-biobased and non-degradable plastics, (bio)degradable polymers offer a number of benefits (Chen et al., 2013). They consistently exhibit better life cycle analyses (LCA) and lower carbon footprints. Recycling is also a possibility, but the amount of knowledge and experience is less than with well-established fossil polymers [and collection and recycling schemes are mostly not in place (yet)]. Recycling, which is the collection, reprocessing, and subsequent use of a plastic materials, is possible due to the ability of thermoplastics to be remolten. “Thermal recycling” is a misnomer because incineration wastes the material apart from capturing its energy, and the carbon released. One can argue that it is the final stage of cascaded use, still the end point is CO_2_ being emitted to the atmosphere.

Due to their characteristics, polyesters make up the largest group of all degradable polymers. Common examples of these biodegradable polyesters are polycaprolactone (PCL), polylactic acid (PLA), and polyhydroxyalkanoates (PHAs) (Khosravi Darani, 2020) as well as PBS and PBAT (polybutylene succinate, polybutylene adipate co terephthalate), with rates of biodegradation and environments where this is the case depending on biopolymer type and properties. Amongst all these polyesters, PHA take a special role, in that they were found to be most readily degradable, not being limited to particular ecosystems. PHAs are formed as granules in the cytosol of many different types of bacteria and extremophile Archaea members. PHA, mainly as its simplest compound, PHB (polyhydroxybutyrate), or more exactly P3PHB, was discovered to be synthesized intracellularly by over 350 species (Kutz, 2011). The agglomeration of PHAs in cells is induced by inhomogeneous growth conditions brought on by nitrogen or phosphorus limitation. PHA granules are created using excessive carbon with the purpose of long-term carbon storage. These biopolymers can be found in nature and are produced naturally by some types of bacteria and other organisms, including *Cupriavidus necator, Chromatium vinosum,* and *Pseudomonas aeruginosa*, and they can be produced through fermentation to make a bioplastics material P3HB generally forms in natural environments. Also, P4HB was found to exist, and several copolymers. The PHA polymer chains can incorporate comonomers when they are present to produce e.g., many odd-numbered fatty acids can serve as 3HV-precursors, including P3HBV (polyhdroxybutyrate-co-3-valerate), in the case of cofed valeric acid e.g., propionic acid, levulinic acid, etc.

**FIGURE 1:**
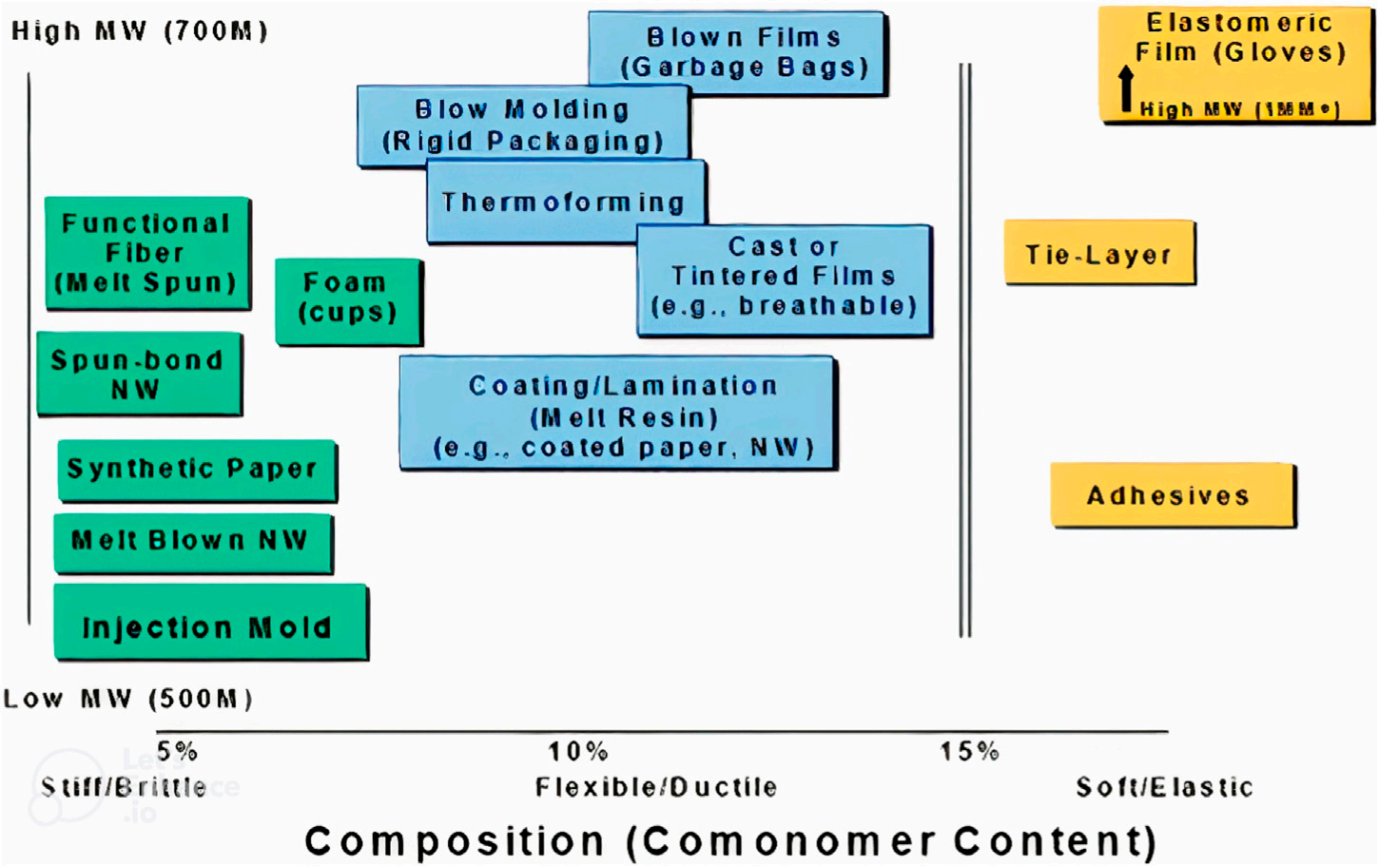
With P3HB copolymers and medium chain length (mcl) PHA, the polyhydroxyalkanoates (PHA) platform is very broad. MW = molecular weight. Source: (Reichardt and Rieger, 2012). Mcl PHA contain 6-14 carbon atoms per monomer.

Polyhydroxy butyrate (P3HB), which can be formed naturally by a number of bacterial microorganisms (Moradi et al., 2019), is the most prevalent and, consequently, the most significant PHA. Transgenic plants were also developed to synthesize PHB. PHA has many more biological functions for the cells that produce it in addition to serving as storage materials (Obruca et al., 2020). P3HB is a substance that occurs naturally in different chain length (Rosetta, 2013). Numerous strains can accumulate it in significant amounts. For instance, *Cupriavidus necator* was found to produce P3HB fractions in its cells that were greater than 75% (by mass) (Holmes, 1985; Vadlja et al., 2016). Apart from sugar and starch, several waste materials can be used as raw material for PHA production, e.g., glycerol. See (Sirohi et al., 2020) for a list of potential feedstocks for the production of P3HB. Despite other waste streams being researched, sugar is still used frequently, comparable to the feedstock of first generation biofuels. Cyanobacteria can produce P3HB when CO_2_ is the only carbon source [100], but their growth rates are slow. Other promising feedstocks for the production of P3HB are acetic acid and formic acid produced from CO_2_ (Vlaeminck et al., 2022), through biological, thermal, catalytic or electrochemical processes. The sole source of carbon and energy for methanotrophic bacteria is CH_4_, which is therefore another promising feedstock to obtain P3HB. The reaction can proceed via either two potential carbon pathways: the serine pathway or the ribulose monophosphate pathway (RMP) (Lemoigne, 1925; Khosravi et al., 2003; Koller, 2014; Khosravi Darani, 2020). P3HB is used, for instance, for drug delivery systems frequently and is biocompatible (Khosravi et al., 2003; Tabandeh and Vasheghani, 2003). As a result, pharmacists use it to produce microcapsules and package materials for cells. Polyhydroxybutyrate can also be used in the food industries and packaging, biodegradable films, non-wovens (diapers, wet wipes) and paper coating, to name a couple (Griffin, 1994). In recent years, P3HB has also been used as blend partner for PLA in filament for additive manufacturing, as well as for various single-use and durable consumer goods like kitchenware. In terms of its properties, P3HB is similar to the common polymer PP, except for higher brittleness. PHA are extremely adaptable polymers that can have their comonomer type, content, and molecular weight changed to achieve a wide range of properties, making them suitable for a variety of processes (see Figure 1).

Common PHA, apart from PHB (P3HB), are PHV, PHHx and PHO, and their copolymers (PHV = polyhydroxyvalerate, PHHx = polyhydroxyhexanoate, PHO = polyhydroxyoctanoate). For an overview of PHA properties, see e.g., (Raza et al., 2018).

PHA are thermoplastics, which makes them mechanically recyclable.

In 1952, Maurice Lemoigne discovered P3HB in the cytosol of *Bacillus Pasteur* (and earlier in *Bacillius megaterium*). That same period saw the clarification of its general formula ( $C_{4}H_{6}O_{2}$ )_n_ (Lemoigne, 1925; Khosravi et al., 2003; Tabandeh and Vasheghani, 2003). The melting point of PHB (160°C–180°C, depending on molecular weight and crystallinity) and its increasing rate of accumulation of P3HB in cells with limited nitrogen and phoshorus supply were two molecular and mass properties that researchers were able to identify after extensive research. First commercialization of P3HB production was attempted by some businesses already decades ago, including ICI (Biopol™), but due to low production rates and expensive downstream processing, these projects were shelved in favor of fossil plastics, which were much more readily available and significantly less expensive to produce. Today, a number of manufacturers offer commercial PHA materials, including PHHx, P3HB, PHO, PHBV, and others, and the industry has started to develop (GO PHA, 2022).

This is due to the increased demand for alternative plastic materials with biobased and/or biodegradable property. (Lackner, 2022). There are few legal requirements for using bioplastics (as opposed to e.g., biofuels, where several jurisdictions have implemented admixing quota), and a significant part of demand is triggered by consumers turning increasingly aware of environmental issues, yet there were several misconceptions about bioplastics in the market.

A variety of carbon source feedstocks can be used to make PHA (Khosravi et al., 2003; Tavares et al., 2004; Kantarci et al., 2005; Bhole et al., 2008; Rahnama et al., 2012), (Sirohi et al., 2020). Both natural gas and synthetic natural gas (biogas, landfill gas, etc), which are carbon-rich substrates, are widely available throughout the world, including distribution infrastructure, with the former being more prevalent in countries with a significant oil industry. The CH_4_ from these feedstocks can be utilized by methanotrophic microorganisms. Methanotrophs can therefore act as a dependable hosts for the synthesis of biopolymers like P3HB. Methanotrophs can be either aerobic or anaerobic, but only the former are significant in terms of technology. Aerobic methanotrophic bacteria can be classified into Type I, Type II, and Type III based on how they take up carbon: Type I uses the ribulose monophosphate (RumP) cycle, Type II utilizes the serine cycle, and Type III deploys the Calvin-Benson Bassham (CBB) cycle. Type I bacteria also rely on the ribulose monophosphate cycle. See (Lackner, 2022; Vlaeminck et al., 2022). The reader is directed to (Lackner, 2018; Van Roijen and Miller, 2022) for a review of biopolymers in general. In short, PHA have a low market share today, on the order of 100,000 tons/year, which is an order of magnitude less than PLA (polylactic acid), and several orders of magnitude less than the commodity plastics in use today for both short-lived and long-lived products, but they see increased interest both from academia and industry and hold great promise to pick up a significant fraction of plastics production volume in the near future, should the boundary conditions be created. This paper aims at contributing to that feasibility by analysing different bioreactor setups for cost-effective and scalable P3HB production. It cannot be stressed enough that scalability is the critical term in a transition to a biobased and biodegradable (and circular) plastics economy.

## 2 Experimental

### 2.1 Bioreactors

This section summarizes reasonable fermenters (bioreactors) for P3HB production by methanotrophs. The traditional fermenter configuration could be a continuously stirred tank reactor (CSTR), with bubble column (BC) and airlift (AL) fermenters being specifically used for gas fermentation processes due to the significant amounts of gaseous substrates, i.e., oxygen (or air) and CH_4_, involved. A loop reactor, which is an advanced AL reactor with external recirculation, is an alternative to a BC or an airlift reactor. A pump is used to facilitate the flow. That pump can be of centrifugal or axial configuration, depending on required flow and head. Provisions are needed to pump the mixed flow (typically 5%–15% of entrained gas in water) with good energy efficiency. As the rate-limiting step, the mass transfer of the poorly soluble CH_4_ and O_2_ in the aqueous phase has been tested and described in various bioreactor embodiments. The solubility values are close to 25 mg/L g of O_2_ in water (at ambient pressure and 30°C) and about 7.5 mg/L of CH_4_ (Zhu et al., 2022) in water (at ambient pressure and 30°C) (Zhu et al., 2022), compared to e.g., CO_2_, which is soluble with 1.25 g/kg of water under the same conditions. Using vectors to increase CH_4_ solubility has been tried (Han et al., 2009). Generally, the concentration gradient and the k_L_a value affect the gas transfer rate (GTR), which is also true for O_2_ and CH_4_. Increasing gas flow and specific energy intake generally help (e.g., a fast stirring rate, and fine spargers). Increased pressure can be used to achieve a higher gradient of gas concentration:

$$GTR=k_{L}a*\left(c{*}_{gas}\;–\;c_{gas}\right)$$

were

GTR = gas transfer rate [mmol/(l*h)]

kLa = volumetric mass transfer rate [h^-1^]

c*_gas_ = saturation concentration of CH_4_ or O_2_ in the medium [mmol/l]

c_gas_ = actual gas concentration of CH_4_ or O_2_ in the medium [mmol/l]

The volumetric mass transfer rate is a product of the mass transfer coefficient k_L_ and the gas-liquid exchange area per unit of liquid volume a (INFORS, 2020).

Thermophilic methanotrophs can result in lower production costs because they require less cooling (Levett et al., 2016), since approx. Half of the energy of the supplied methane ends up in biomass and half is metabolized, but a balance must be found for sufficient gas solubility in the aqueous medium at higher temperatures. Particularly when low cell densities are encountered, downstream processing is a cost driver for P3HB production, where cell densities are lower than e.g., with sugar as substate.

### 2.2 Vertical tubular loop bioreactor (VTLB) with forced liquid flow

Adding a pump to the second loop (downcomer, i.e., the return loop) of the airlift reactor can increase the liquid flow rate for higher mass transfer. Loop reactors can be essentially vertical or horizontal in configuration. The performance of a Vertical Tubular Loop Bioreactor (VTLB) using a forced liquid flow model was reported by Yazdian et al., (2010) and Khosravi Darani, (2020). In general, the main costs of biomass production from aerobic microorganisms are chemicals (macro-and micronutrients) and energy (oxygen production and dissipation of O_2_ and CH_4_), apart from cooling energy. The special properties of VTLB make it a suitable fermenter configuration for the production of biomass such as P3HB or other bulk materials such as SCP (single-cell protein), another product by methanotrophs of commercial interest, as feed and food ingredient. Design and control studies for VTLB were carried out in the above work. In this study, a vertical loop bioreactor was used at laboratory scale. It was made of glass and equipped with a dissolved methane detector (Anabtawi et al., 2003).

**FIGURE 2:**
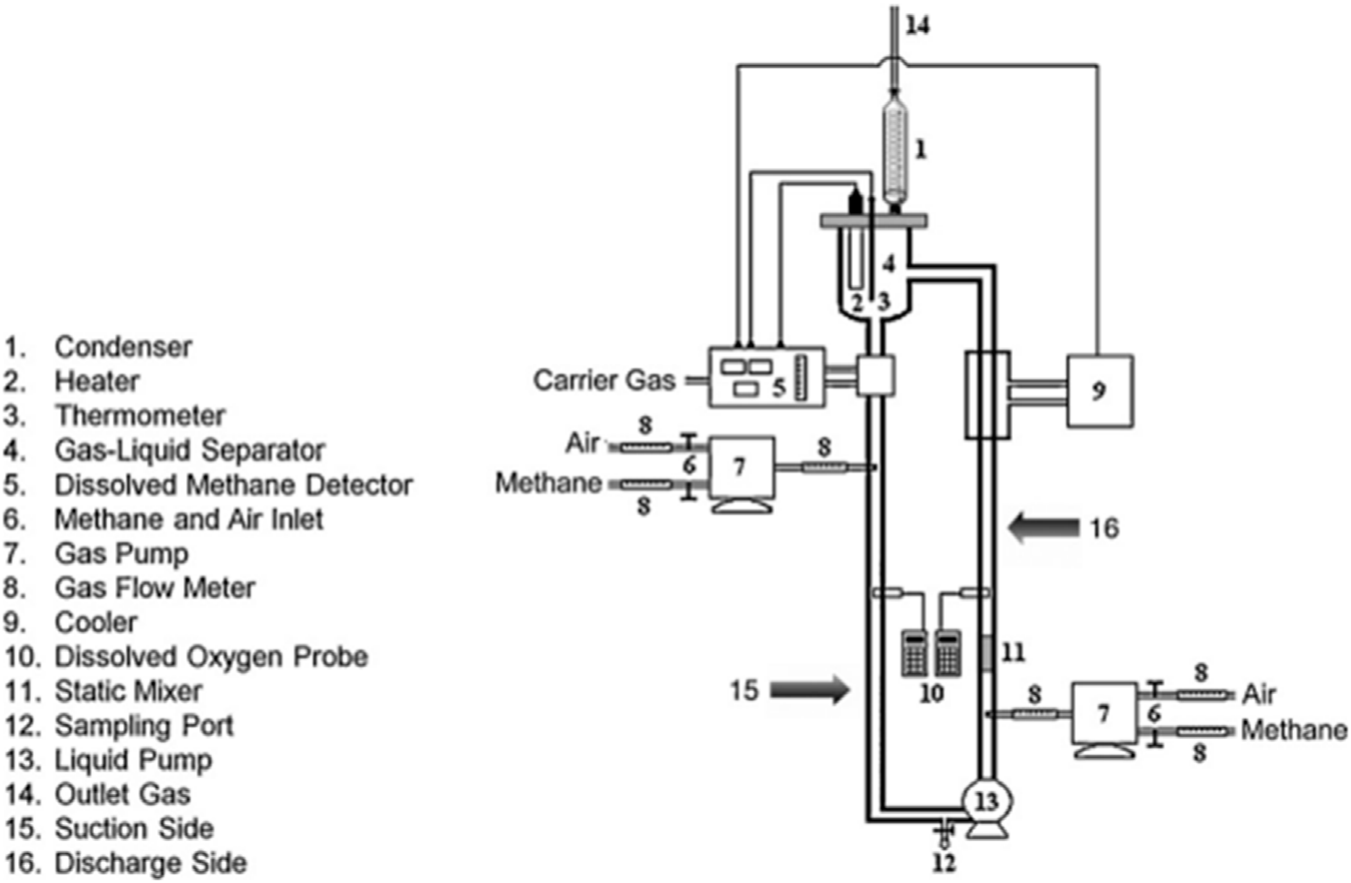
A schematic representation of the forced-liquid vertical tubular loop bioreactor (VTLB) (Rahnama et al., 2012), (Khosravi-Darani et al., 2020). The flow speed in the VTLB was varied between 0.35 and 0.035 m/s, and the flow regime was found to be turbulent. An advantage for the vertical setup of the bioreactor is the lower space requirement compared to an HTLB (see below).

Figure 2 shows a schematic illustration of the VTLB. The VTLB is constructed from a glass pipe with a single-wall-loop (Rahnama et al., 2012). The essential traits are shown in Table 1. Natural gas was supplied and dispersed, along with oxygen, through a combined pierced tube within the vertical section using a gas pump that supplied natural gas (mainly methane; 90% by volume). Between 0.2 and 0.5 vvm (vessel volumes per minute), were used, and the gassing rate was adjusted for maximum productivity. The CH_4_:O_2_ ratio was kept constant at 1:1 (by volume). Gas bottles containing 99.9% (by volume) of O_2_ were used to provide the oxygen.

**TABLE 1:** Forced-liquid vertical loop bioreactor (VTLB) with key geometric and operational parameters (Rahnama et al., 2012).

Descriptions	Unit	Value	Comment
Riser diameter, $D_{r}$	[m]	0.03	
Downcomer diameter, $D_{d}$	[m]	0.03	
Separator diameter, $D_{s}$	[m]	0.11	
Liquid level in separator, $h_{s}$	[m]	0.10	
S = $V_{s}/V_{b}$	[-]	0.61	separator to bioreactor volume ratio
H/D	[-]	67	height of bioreactor, [m], to diameter of the bioreactor [m]
Holes size in sparger, $D_{O}$	[mm]	0.1	
Number of holes in the distributor, N	[-]	6	One for natural gas, one for O_2_

A magnetic pump was deployed to regulate the movement of fluid. Natural gas has explosive limits of approx. 5%–15% in air and of 5%–60% in oxygen, so that type of pump was chosen to accommodate those properties for safe handling in the lab environment. The riser (discharged) part of the VTLB, which was about 0.1 m in length, contained a static mixer (Komax type) that was used to mix the fluid. O_2_ and natural gas were injected through two tubular spargers, one on either side, with six holes each, prior to (upstream of) the static mixer. A glass coil for condensing waterwas placed on top of the fermentation vessel to recover lost medium (evaporated water). Additionally, the loop had a jacket on one side to provide cooling for theexothermic process. A thermocontroller at the upper part of the VTLB was connected to a temperature loop controller that kept the temperature at 30 ±0.5 ℃ . By using a pH probe, the system’s pH was managed (Rahnama et al., 2012). The fermentation broth was degassed of the formed CO_2_.

Figure 2 shows a schematic illustration of the experimental setup with the vertical loop reactor.

The flow speed in the VTLB was varied between 0.35 and 0.035 m/s, and the flow regime was found to be turbulent.

An advantage for the vertical setup of the bioreactor is the lower space requirement compared to an HTLB (see below).

### 2.3 Horizontal tubular loop bioreactor (HTLB) with forced liquid flow

An alternative embodiment for a loop reactor is an HTLB (Bouaifi et al., 2001), where the largest section of the tubular loop is arranged horizontally, with only a small vertical part. (Bouaifi et al., 2001).describes a single-walled, forced-liquid horizontal tubular loop bioreactor (HTLB) with a methane detector, which was used as basis for the current work. This bioreactor’s geometry was altered, and experiments were conducted using a model with a diameter to height ratio of 3:40 and a total height of 220 cm. Via the horizontal part, the natural gas and the O_2_ were distributed by a bore tube through a gas pump in diverse regions. Two Komax static mixers in the horizontal part were used to produce motion (vortex-type flow pattern). Based on the dimensions of HTLB and liquid flow speed, which was 0.24 m/s, the flow inside of it was turbulent.

For medium pumping (lower head required for the pump), a horizontally constructed bioreactor uses less energy. This is the main advantage over a vertical setup.

### 2.4 Airlift bioreactor (ALB)

Airlift bioreactors (ALB) used for gas fermentation have a number of benefits. On the one hand, the design of the bioreactor is straightforward and uses little energy because there is a lack of mechanical parts to can cause shear stress and pressure drop. A reduction in the bulk density of the fluid in the riser part contrasted to the down-comer zone happens when in draught-tube airlift reactors, air is introduced into the medium from a sparger, which is placed at the bottom of the central tube (Khosravi-Darani et al., 2020). This type of bioreactor is frequently used to produce human antibodies from animal cell cultures as well as beer and vinegar from different microorganisms like yeast, bacteria, and fungi. The advantages of an airlift bioreactor include a high mass transfer coefficient and the ability to load the suspension with solid particles (Tavares et al., 2004). They can be compared to bubble column (BC) bioreactors, which have a less clearly defined flow profile. In order to achieve a higher conversion rate of the gaseous feedstock, ALB is hence preferred over BC.

### 2.5 Bubble column (BC) bioreactor

In many different processes, including those in the metallurgical, petrochemical, and biochemical industries, bubble column (BC) reactors are commonly employed (Kantarci et al., 2005). The low-complexity device (in our case, a bioreactor) comprises of a tubular vessel and a gas distributor at the base of the reactor. A sparger injects gas into the standing liquid or solid-liquid column in the form of bubbles, which rise through the column and mix the medium while releasing energy. As a result, bubble columns are frequently used in biochemical processes like fermentation and biological sewage treatment (Luo et al., 1999; Shimizu et al., 2000; Bhole et al., 2008) due to their ease of setup and operation. Numerous parameters have been examined in the past, including gas holdup (Li and Prakash, 2000; Anabtawi et al., 2003), (Vinod and Kumar, 2011), bubble characteristics (Li and Prakash, 1999; Li and Prakash, 2000; Ruzicka et al., 2001; Buwa and Ranade, 2002; Lapin et al., 2002; Schäfer et al., 2002), reports on computational fluid dynamics and flow regime (Degaleesan et al., 2001; Li and Prakash, 2001; Li and Prakash, 2002; Michele and Hempel, 2002; Dhotre et al., 2004; Thorat and Joshi, 2004; Vinod and Kumar, 2011), studies of measurements of heat (local and average) (Lin and Wang, 2001; Behkish et al., 2002; Chen et al., 2003; Verma and Rai, 2003), and mass transfer (Pino et al., 1992; Lefebvre and Guy, 1999; Krishna and Van Baten, 2003; Maalej et al., 2003; Vandu and Krishna, 2004; Vinod and Kumar, 2011). Arcuri et al. (Arcuri et al., 1986) conducted extensive research on the production of thienamycin (thienpenem, CAS no. 59995-64-1), one of the most powerful naturally occurring antibiotics, in *Streptomyces cattleya*, in a bubble column bioreactor. The production of recombinant antibodies was significantly increased by the hybridoma cells culture method, which is used to create large quantities of monoclonal antibodies (Arcuri et al., 1986). Son et al., (2000) developed a new type of bubble column bioreactor to manufacture taxol (paclicataxel, CAS no. 33069-62-4), an alkaloid useful for treating cancer, in various bubble column bioreactors to characterize cell growth.

Shiao et al., (2002) used the hairy roots of *Arabidopsis thaliana* (also known as Thale cress, a small weed and popular model organism) in a bubble column reactor.

**FIGURE 3:**
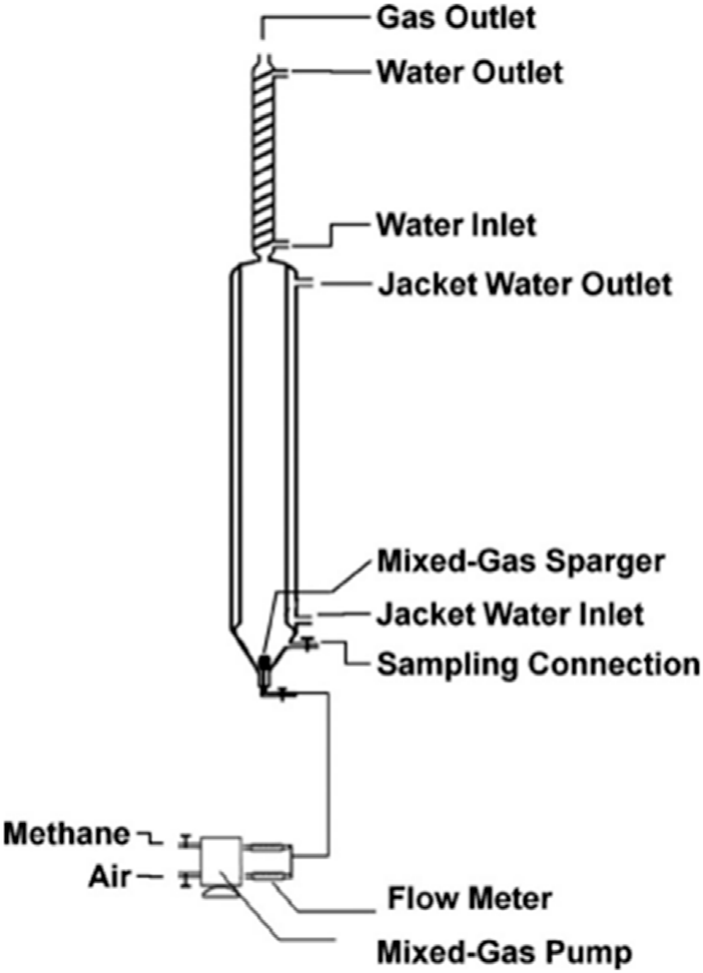
A schematic diagram of a lab scale bubble column bioreactor for methanotrophs (Yazdian, 2005).

The schematic of a bubble column bioreactor is shown in Figure 3, for a laboratory-scale application.

Compared to loop reactors (VTLB, HTLB), all energy for liquid mixing and transport is supplied by the compressed gasses on ALB and BC, which renders them less energy-efficient due to energy losses in the gas compression. They are mechanically less complex, though.

### 2.6 P3HB production in bioreactors by methanotrophs

The methanotrophic strain *Methylocystis hirsuta* (DSMZ 18500), which had been obtained from the DSMZ collection in Germany, was used for the fermentation trials described here (Rahnama et al., 2012). P3HB is accumulated by this gram-negative, aerobic strain through the serine pathway. Type II methanotroph (Rahnama et al., 2012) describes its characteristics.

### 2.7 P3HB production in a bubble column bioreactor

Yazdian et al. (2005) (Rahnama et al., 2012), (Shahhosseini and Khosravi, 2003) used a 1 L glass column with 51.0 cm height, 5.0 cm inner diameter and 200 mL working volume. The temperature and the pH of the reactor were adjusted and maintained at 30 ℃ and 7.0. In order to prevent water loss from the medium, on the top of the column, a condenser was installed. With a sparger which had 40 fine orifices, natural gas entered through the sparger line together with oxygen (for industrial operation, premixing the two gasses is not recommended, to avoid the formation of an explosive atmosphere). The temperature and pH were set by a heat controllable water bath and 1.0 N HCl/NaOH, respectively (Rahnama et al., 2012). Firstly, the selected 20 mL of shake-flask cultures were inoculated into 180 mL of fresh culture and incubated at 30 ℃ under regular natural gas and air aeration. Secondly, the cells were harvested by centrifugation at around 5,000 rpm. Subsequently, in the limitation of nitrogen, the cells were re-suspended in the medium (Yazdian et al., 2010) to accumulate P3HB, and because of this two stage cultivation approach this process is semi-continuous. Depletion of nitrogen caused the P3HB content to increase sharply to 62.3% of cell dry weight at 52 h (the P3HB synthesis started after 30 h).

### 2.8 P3HB production in an air lift bioreactor

In an airlift bioreactor, Tavares et al., (2004) studied production of P3HB by *Cupriavidus necator* DSM 545. All the experiments were done in an airlift bioreactor with a working volume of 10 L which was equipped with pH probe and temperature control (Khosravi Darani, 2020). To compare the airlift bioreactor with a stirred-tank fermenter (7 L of working capacity) at the lab scale, experiments were conducted. The internal diameter of the stirred-tank fermenter was 18.8 cm. It was also equipped with flat blade turbines with baffles (diameter of 7.5 cm), and controllers for pH and temperature monitoring. At the bottom of the stirred-tank, a stainless-steel sparger was located. In P3HB production, there is the above-described two-stage method “feast and famine” for cell cultivation, which was also applied here. In the first step, the stock culture was incubated for 15 h under 30 ℃ and 250 rpm. In the subsequent step, 10% (v/v) inoculation broth was added to the medium and incubated (15 h). P3HB accumulation was induced by the limitation of ammonium. The cell concentration was determined by measuring the CDM (cell dry mass) after centrifugation at 9220 *×* g for 10 min at 4 ℃ and pursuant filtration using a 0.45 µm membrane and drying until constant mass was established (Khosravi-Darani et al., 2020). Some definitions, such as residual biomass ( $X_{r}$ ) were determined as total cell mass minus P3HB mass, the ratio of P3HB (P3HB%) to total cell mass, and pO_2_, the partial pressure of oxygen, were measured. With GC (Shahhosseini and Khosravi, 2003) the P3HB concentration was quantified. The inlet gas composition and the outlet gas flow were analyzed by paramagnetic analyzers (O_2_) and mass flow meters (Tavares et al., 2004) to establish the mass balance.

During the accumulation phase, P3HB production was carried out by six different runs through six different aeration values.

### 2.9 P3HB production in a forced-liquid vertical loop bioreactor

Yazdian et al., (2010) and Khosravi Darani, (2020) performed research in a forced-liquid vertical loop bioreactor (VTLB) (Forret et al., 2003), whose schematic is shown in Figure 2. Based on Yazdian et al., (2010), the flow regime with the highest mass transfer coefficient was found there. Parameters such as H/D, $U_{SL}$ and $U_{SG}$ define the height to diameter ratio of VTLB, superficial liquid velocity [m/s] and superficial gas velocity [m/s], respectively (Khosravi-Darani et al., 2020). They can be explained as follows:

H/D, the height to diameter ratio, is the most relevant geometric parameter. We found that min. 3 cm diameter of the vertical and horizonal pipes were needed for stable operation, which was determined by the ratio of diameter to bubble size.

$U_{SL}$ (Superficial liquid velocity): a hypothetical (artificial) flow velocity calculated as if the given phase or fluid were the only one flowing or present in a given cross-sectional area (Khosravi-Darani et al., 2020).

$U_{SG}$ (Superficial gas velocity): an estimate of how quickly a fluid passes through an object (e.g., a pipe) or a porous medium

**FIGURE 4:**
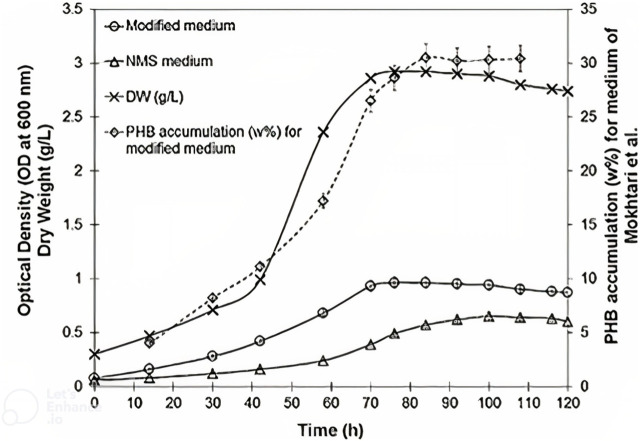
Comparison of NMS (nitrate mineral salts) and modified medium [Mokhtari et al. (Koller et al., 2013)] for cell growth and P3HB accumulation of *Methylocystis hirsuta* in a bubble column bioreactor (Rahnama et al., 2012), (Khosravi Darani, 2020). For the modified medium of Mokhtari et al., see (Koller et al., 2013).

In each experiment, S (separator to bioreactor volume ratio), H/D, $U_{SL}$ and $U_{SG}$ were set and fixed in an ideal range. An instrument for measuring dissolved methane monitored the distribution of methane through a tube in a vertical section (Khosravi Darani, 2020). The VTLB had a 51.6% (w/w) P3HB accumulation in terms of cell dry mass (CDM). For the first time, P3HB production from natural gas was carried out in both a vertical loop bioreactor and a bubble column bioreactor using P3HB accumulation by microorganisms like *M. hirsuta for direct comparison*. It was discovered that under optimum processing circumstances and using this bacterium (*M. hirsuta*) for P3HB production, the cells accumulate just over said 51.6% cell dry mass of P3HB in the VTLB (Figure 4). The development of this P3HB bioprocess uses natural gas as an affordable substrate (Khosravi Darani, 2020), but could also have been done on biogas or any other renewable CH_4_.

The different growth systems can be compared as follows (Table 2).

**TABLE 2:** Comparison of lab-scale gas fermentation systems for P3HB production. Compare (Khosravi-Darani et al., 2020).

Reactor	Continuously stirred tank reactor (CSTR)	Forced-liquid vertical loop bioreactor (VTLB)	Forced-liquid horizontal tubular loop bioreactor (HTLB)	Airlift (AL) fermenter	Bubble column (BC) fermenter
Methane conversion	medium	high	high	high	high
Energy efficiency	Lowest	high	highest	medium	medium
Cooling	Difficult at large scale	best	best	good	good
k_La_ value	0.0056 s^−1^	0.034 s^−1^	0.037 s^−1^	0.0482 s^−1^	0.028 s^-1^
Productivity	0.14 g/(L·h)	1.0 g/(L·h)	0.786 g/(L·h)	0.15 g/(L·h)	0.18 g/(L·h)
					
References	Kianoush (2020)	Zúñiga et al. (2011)	Jones (2015)	Khanna and Srivastava (2005)	Larsen (2002)

Based on the measured productivity, the VTLB, was found to be most advantageous.

### 2.3 Simulation of fermenters of methanotrophic P3HB production

#### 2.3.1 Simulation of an airlift bioreactor

In order to simulate the flow (Roy and Joshi, 2008; Mousavi et al., 2010) and hydrodynamic properties in bubble columns, stirred-tank (Roy and Joshi, 2008; Wang et al., 2009; Mousavi et al., 2010; Amini et al., 2013), airlift, and membrane bioreactors, computational fluid dynamics (CFD) has developed into a useful and adaptable tool. This simulation approach is gleaned from governing equations of momentum, energy, and continuity for each phase. There are many benefits to using CFD simulation, such as that the geometry of the column and scale impacts are taken into account automatically, the experimental inaccessibility to several locations in the system is alleviated, and turbulent multiphase streams can be studied. Also, high costs of traditional laboratory experiments, where CFD simulation is a cost-effective tool, are avoided. The time for experiment completion can also be reduced. Ergo, CFD simulations can ease the investigations and evaluation of characteristics of flow regimes in novel setups like bioreactors of VTLB and HTLB configuration. To confirm and ensure the validity of the modeling and possibly adapt it to reality, a concurrent approach involving modeling and experiments is advised, as done in this work.

Computational fluid dynamics (Huang et al., 2010; Luo and Al-Dahhan, 2010; Bannari et al., 2011; Luo and Al-Dahhan, 2011; Šimčík et al., 2011) is one of the most useful and powerful tools for determining fluid dynamics properties, including shear stress, gas holdup, flow dynamics, and mass transfer in airlift and bubble column bioreactors. In recent decades, the features and capabilities of modeling software have evolved significantly and are used to evaluate fluid dynamics and designs of new bioreactor models.

The optimization of the kinetic parameters was carried out in a study by Tavares et al., (2004), who modeled cell growth and bioplastics accumulation steps for P3HB production. Leaf and Srienc, (1998) proposed a model and studied the production of P3HB in a bioreactor. Their model comparison is based on Eq. 1.

$$\frac{d\left[PHB\right]}{dt}=R_{synthase}-\mu_{s}PHB$$ (1)

where $R_{synthase}$ displays the rate of enzyme-catalyzed reactions and $\mu_{s}$ shows the specific growth rate (Tavares et al., 2004).

The feed was made up of glucose and fructose. Since both of them changed and are converted to glucose-6-phosphate, glucose was taken for granted to be the only substrate in the simulation. In this study, a two-phase stream Eulerian model was used. A complex multiphase model is a Eulerian model. It is comparable to the Lagrangian model, with the exception that it deploys a fixed reference grid as opposed to the moving grid of the Lagrangian model.

The continuity equation is written as:

$$\frac{\partial }{\partial t}\left(\alpha_{j}\rho_{j}\right)+\nabla .\left(\alpha_{j}\rho_{j}\vec{u_{j}}\right)=0$$ (2)

Where $\alpha_{j}$ shows the fraction of volume, $\rho_{j}$ describes density, and $\vec{u_{j}}$ defines the average velocity of phase j.

**FIGURE 5:**
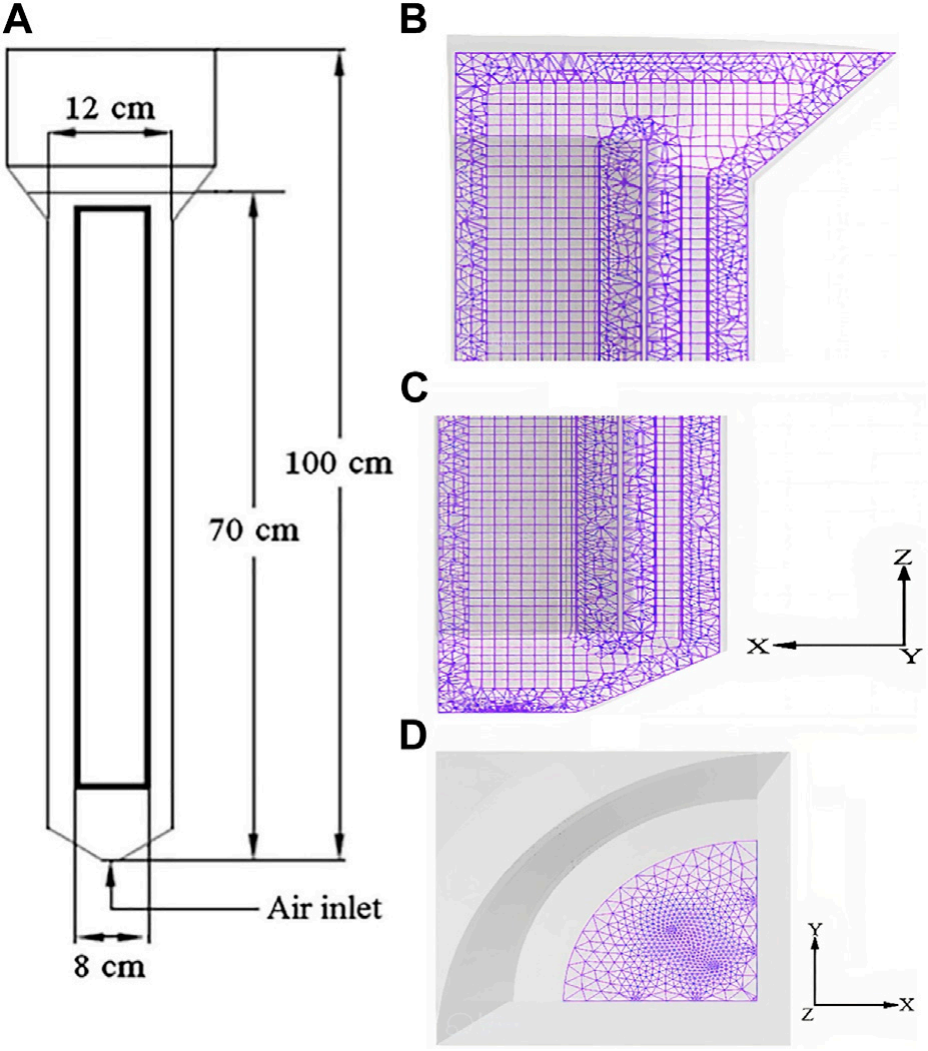
**(A)** Schematic diagram of the simulated bioreactor (Mavaddat et al., 2014), and the generated mesh of the bioreactor at **(B)** upper side view, **(C)** lower side view, and **(D)** bottom cross view (Sparger).

Figure 5 shows the schematic of the bioreactor which is investigated by Tavares et al., (2004). Due to the symmetry of the vessel, a section of thebioreactor can be used as the simulation’s base. Fluent 6.3.26 and GAMBIT 2.3.16 were utilized for simulation and creating the geometry and grids. For a good performance of the modelled bioreactor, the flow pattern around the diffuser (sparger) is essential. Therefore, to ensure convergence of the model, the geometry was meshed using the size function method (Figures 5B–D). The SIMPLE method of pressure–velocity coupling was applied, compare (Khosravi-Darani et al., 2020). Relaxations factors, including body force, pressure, momentum and density, which are equal to 1, 0.3, 0.7, and 1, respectively, were incorporated. The liquid phase in the mode was a combination of water, glucose, H^+^, CO_2_, and P3HB monomers. As mentioned above, P3HB is a biopolymer which exists in the cytoplasm, but in the Fluent software, which was used in this study, P3HB-comprising cells possess molecular weight and density of 86.09 g/mol and 1,250 kg/m^3^ (Doyle et al., 1991), respectively.

#### 2.3.2 Simulation of a tubular loop bioreactor

For the production of biopolymers such as P3HB from natural gas, loop bioreactors are particularly suitable due to their special hydrodynamic properties. They can be approximated by a plug flow pattern, where high conversion efficiencies of the fed gasses are feasible. An important hydrodynamic property in a loop bioreactor is gas holdup. The determination of gas holdup is important because of its effects on gas residence time, oxygenation, heat and mass transfer, fluid flow rate, and mixing (Aminabhavi et al., 1990). Several phases are common in the processing industries. Methods and models must be able to address these multiple types of flow. There are lots of complexities for CFD simulations in two-phase flows. By combination of readily available fractional factorial matrices or orthogonal arrays (OA), the experiments which led to an optimal set of performance characteristics were minimized. Taguchi design is used broadly in several experimental methods to optimize the vital parameters of any process (Khosravi et al., 2004). OA are able to minimize the experimental iterates. ANOVA can predict the impact of a factor on characteristic properties (Khosravi-Darani et al., 2013). With an algebraic multi-grid method and a point implicit (Gauss-Siedel) linear equation solver, the resulting scalar system of equations is solved. In order to solve prevention equations for chemical species, the software predicts the local mass fraction of ones, the software estimates the mass transfer fraction of each chemical species, $Y_{i}$ . This conservation equation takes the following form:

$$\frac{\partial }{\partial t}\left(\rho \;Y_{1}\right)+\nabla .\left(\rho \;\vartheta \;Y_{1}\right)=\nabla .i+R_{i}S_{i}$$ (3)

$R_{i}$ is defined as the net rate of production of species i by chemical reaction, and $S_{i}$ describes the rate of creation by addition from the dispersed phase plus any user-defined sources (Khosravi-Darani et al., 2020). An equation of this form will be solved for N −1 species, where N is the total number of fluid phase chemical species which are present in the system. Because the mass fraction of the species must sum to unity, the $N^{th}$ mass fraction is determined as one minus the sum of the N −1 solved mass fractions (Khosravi-Darani et al., 2020). Selecting $N^{th}$ species with massive mass fraction, such as $N_{2}$ while the oxidizer is air, led to minimize the numerical error.

$J_{i}$ arises because of the concentration gradients. FLUENT utilizes the weaken approximation, under which the diffusion flux can be written as:

$$J_{i}=-D_{i,m}\nabla Y_{i}$$ (4)

**FIGURE 6:**
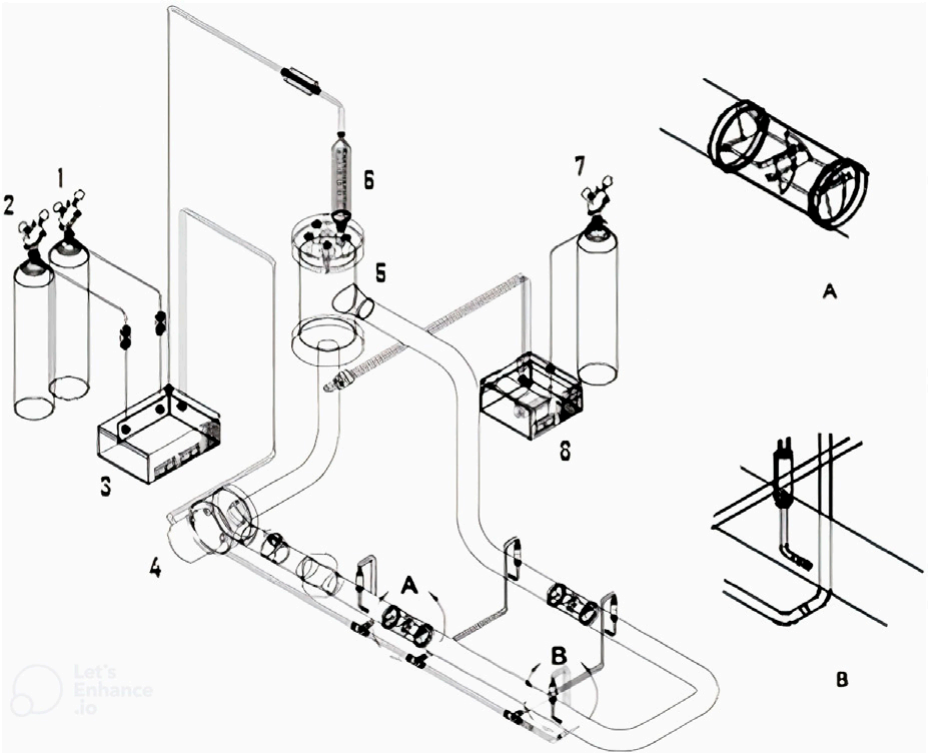
Schematic diagram of the forced-liquid horizontal tubular loop bioreactor used for the experiments: (1) natural gas supply; (2) air supply; (3) gas pump; (4) liquid pump; (5) gas–liquid separator; (6) condenser; (7) helium supply; (8) dissolved methane detector. Magnified views of the (A) static mixer and (B) sparger (Mousavi et al., 2009). Reproduced from (Khosravi-Darani et al., 2020).

**FIGURE 7:**
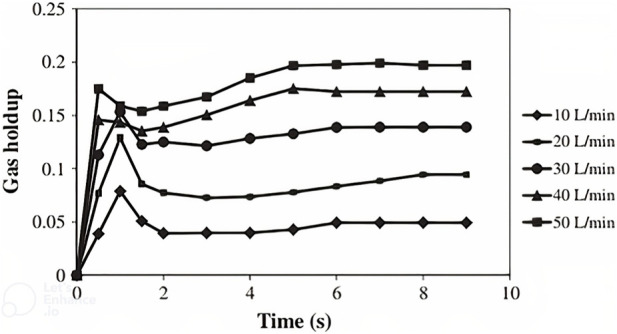
Gas holdup vs. time for five different aeration rates (Mousavi et al., 2009).

Here D_i,m_ is the diffusion coefficient for species i in the combination m. A commercial grid-generation tool (GAMBIT 2.2) was used in this study to create the geometry and grids. It is crucial to deal with the governing equations over the solution amplitude using a large enough number of computational cells. As previously mentioned, some simplifications were considered in the account in the simulated geometry for this study, including; (a) neglection of the impact of the liquid tube and separator part of the bioreactor; (b) a simple structure for the mixers; and (c) Just four of them were considered to be active holes in the spargers. The static mixer may be eliminated by using a well-designed sparger on the suction side. Four of the sparger’s jet holes were considered being active (Figures 6, 7). The velocities were coupled and solved by phases, but separately. The multi-grid block algebraic scheme was employed. Additionally, a pressure modification equation that used total volume continuity rather than mass continuity was created. The phase continuity equations provided the volume deductions. Three mesh sizes were examined. Time was recorded meticulously; it was 200 s. In Table 1, the comparison of data generated at 200 s for those three grid sizes is visible. Case 2 was chosen as the optimum mesh size according to the accuracy of the resulting data. Despite the fact final volume fractions and velocities for both cases 1 and 2 were similar, number 2 was chosen for simulation because of the smaller number of meshes (Mousavi et al., 2009).

Gas holdup in 10, 20, 30, 40, and 50 L/min aeration rates were obtained by simulation and were compared with the results of various correlations for gas holdup in bubble columns and airlift reactors.

#### 2.3.3 Modeling principles

a) Residual biomass is produced from ammonia and glucose, respectively. The concentration of nitrogen and glucose is important to determine the growth rate and the nitrogen depletion (b) P3HB is produced from glucose during non-growth phase. Excessive nitrogen concentration is applied as an inhibitor which is associated with P3HB synthesis. (c) One part of glucose is converted to ${CO}_{2}$ and several metabolites. (d) Formerly conducted kinetic analysis was performed to measure the basic model parameters. (e) Two growth rate correlations with substrate concentrations (C, N) have been examined: “double-Monod” equation according to Megee et al., (1972) and Mozumder et al., (2015) correlation for double substrate limited growth. Specific non-growth associated P3HB production:

$$a_{n=}\;a_{\max}\left(\frac{s_{n}}{s_{n}+k_{as}}\right)\left(\frac{k_{in}}{N_{n}+k_{in}}\right)$$ (5)

#### 2.3.4 Simulation of P3HB in fed batch culture

In the fed-batch strategy, keeping the concentration of a particular substrate at a minimum level may prevent undesired effects. Without a sophisticated feeding strategy, the system cannot achieve high productivity. Hence, (Shahhoseini and Jamalzadeh, 2006), designed a mathematical model to simulate production of P3HB in a fed batch system. It is completely important to choose the best kinetic design for bacterial growth, for selecting the best parameter set. Experiments were carried out by Suzuki et al., (1986) for P3HB production by *Pseudomonas extorquens* in fed-batch culture. There are several kinetic models investigated by researchers to select the best and the most effective approach. An optimization method has been used to predict and determine model parameters:

$$\delta^{2}=\frac{\sum {\left(model\;data-experimental\;data\right)}^{2}}{degree\;of\;freedom}$$ (6)

The difference between experimental data and estimated parameters in this equation is the degree of freedom. In an investigation on 51 bacterial strains by Suzuki et al., (1986), they selected *P. extorquens* and methanol concentration was kept between 0.2–0.5 g/L (Suzuki et al., 1986). Methylotrophic bacteria are an alternative option to methanotrophic ones, but they require the more expensive feedstock methanol, which is easier to ferment because of its excellent water miscibility. Methanol is also accessible via renewable resources.

The Monod model, a popular mathematical model for the growth of microorganisms, was found not be appropriate for use in this procedure. There are four kinetic models that could fit the experimental data, including growth rate against methanol concentrations that were researched. In Table 2, the kinetic equations were listed. An optimization program was developed to calculate the values of $\delta^{2}$ for each model (Table 3; Table 4). According to these results, the Haldane model leads to the minimum value for $\delta^{2}$ . The Haldane equation has been used extensively to describe substrate inhibition kinetics and biodegradation of inhibitory substrates (Sonnad and Goudar, 2004).

**TABLE 3:** Selected kinetic equations for P3HB production modeling and predicted parameters for the kinetic models.

	Equation	$\mu_{m}\left(h^{-1}\right)$	$K_{s}\left(\frac{g}{L}\right)$	$K_{i}\left(\frac{g}{L}\right)$	$\delta^{2}$
Haldane	$\mu =\frac{\mu_{m}\;s}{\left(K_{s}+s\right)\left(1+\frac{s}{K_{i}}\right)}$	0.2556	0.0251	10.4854	2.75 $\times 10^{-4}$
Edwards 1 [97]	$\mu =\frac{\mu_{m}s}{K_{s}+s}\exp\left(-\frac{s}{K_{i}}\right)$	0.2334	0.0149	19.784	4.1 $\times 10^{-4}$
Edwards 2 [97]	$\mu =\mu_{m}\left(\exp\left(\frac{s}{K_{i}}\right)-\exp\left(\frac{s}{K_{s}}\right)\right)$	0.2304	0.0469	20.16	3.78 $\times 10^{-4}$

**TABLE 4:** Estimated parameters for Mulchandani’s model [98].

	$\mu_{m}\left(h^{-1}\right)$	$K_{sr}\left(\frac{g}{L}\right)$	$S\left(\frac{g}{L}\right)$	N	$\delta^{2}$
Equation					
$\mu_{i}=\mu_{m}\frac{s}{K_{sr}+s}\;\left[1-{\left(\frac{s}{s_{m}}\right)}^{n}\right]$	0.9357	0.0726	6.9379	0.1345	0.0012

#### 2.3.5 Fed-batch model

Mass balance equations:

Mass balance for substrates and the product were considered and the Haldane kinetic model was added onto the equations.

Mass balance for residual biomass:

$$\frac{{dX}_{r}}{dt}=\left(\frac{\mu_{m}S}{\left(K_{s}+S\right)\left(1+\frac{S}{K_{i}}\right)}-\frac{1}{V}\frac{dV}{dt}\right){.X}_{r}$$ (7)

Methanol balance:

$$\frac{ds}{dt}=-K_{1}\frac{{dX}_{r}}{dt}-K_{2}X_{r}+\frac{F_{m}}{V}-S\;\frac{dV}{Vdt}$$ (8)

Nitrogen source mass balance:

$$\frac{{dS}_{n}}{dt}=K_{3}\frac{{dX}_{r}}{dt}+\frac{F_{N}}{V}-S_{N}\frac{dV}{Vdt}$$ (9)

Product mass balance:

$$\frac{dP}{dt}=-K_{4}\;\frac{{dX}_{r}}{dt}-K_{5}X_{r}-P\;\frac{dV}{Vdt}$$ (10)

Total feed rate to the bioreactor:

$$\frac{dV}{dt}=Feed\;rate-Volume\;loss$$ (11)

According to Eq. 6, volume loss is sampling volume/h + rate of volume loss of evaporation.

#### 2.3.6 Simulation results of the first method

In the P3HB production phase, no nitrogen source was fed into the reactor. Shahhoseini and Jamalzadeh, (2006), presented an optimization program to estimate the values of the K1-K5 parameters. In each repetition of the optimization procedure, Eqs 2–6 were solved by employing a program.

#### 2.3.7 Simulation results of the second method

Suzuki et al., (1986) applied the pH-stat strategy in the 1st phase, and ammonia was continuously fed in the 2nd phase. For the first experiment, the feeding rate was set at 0.02 $\frac{{g\;NH}_{3}}{h}$ and for the second, this value was set to one-fourth (Shahhoseini and Jamalzadeh, 2006).

The specific production rate of P3HB can be described as follows:

$$q_{PHB}={\left(q_{PHB}\right)}_{0}\exp\left(-k_{d}t\right)$$ (12)

where $q_{PHB}$ is the specific production rate of P3HB, t is the time when complete consumption of the nitrogen source occurs, ${\left(q_{PHB}\right)}_{0}$ is the value of $q_{PHB}$ at t = 0 and $k_{d}$ is the specific reduction rate of P3HB.

The values of $k_{d}$ and ${\left(q_{PHB}\right)}_{0}$ were estimated by employing data from four experiments. The meaning of $q_{PHB}$ is the same as $K_{5}$ in Eq. 7.

## 3 Discussion

In this work, several different types of fermenters are compared with regard to gas fermentation, which is used to produce P3HB from methane as the only energy and carbon source. In order to accomplish this, experiments and simulations of models were carried out in an effort to identify the most productive and efficient production technology. The cost of the substrate and the recovery process was found to be up to ten times higher than with conventional polymers in (Shahhosseini and Khosravi, 2003), which highlights how important a low cost feedstock and efficient downstream processing are to cost.

Hence, for cost-effective P3HB production, apart from an optimized fermentation process, an environmentally friendly, low-energy consuming extraction process needs to be deployed. Coming back to the fermentation process, waste streams as feedstocks can be more sustainable than primary agricultural products, and the authors find conversion of the former into gaseous substrates advantageous over enzymatic cleavage into sugars. As production strains, naturally occurring microorganisms can be used, or genetically engineered ones (Heijstra et al., 2017). Metabolic engineering for non-food target products can provide novel target compounds and/or better yields. For a cost-effective medium, sludge filtrate from sewage treatment was suggested (Lee et al., 2022). Another important strategy is medium recycling from biomass harvesting.

Amongst the decisive factors for fermenter selection, scalability is of pivotal importance. Gas fermentation bears its own challenges in scale-up (Heijstra et al., 2017). Commercial gas fermentation operations are still limited today; Companies Coskata, INEOS Bio, and LanzaTech have built and operated pilot plants (Heijstra et al., 2017) around the acetogens platform. For instance, Coskata made ethanol from synthesis gas. Ineos also deployed synthesis gas (made from biomass and waste gasification) to make ethanol, as well as Lanzatech, who have recently announced progress to produce ethylene (GLOBE NEWSWIRE, 2022).

Several companies are developing technology to make single-cell protein (SCP) from methane using aerobic methanotrophs (Ritala, 2017).

As for P3HB from methane, that process has not yet been commercialized, despite several companies working on this endeavour. For a vision of a methanotroph-based bio-refinery, see (Strong et al., 2016).

## 4 Conclusion

A high-throughput, active biomass process should be used to product P3HB. Due to their long gas residence times and possible use of low-cost, carbon-rich substrates, loop bioreactors are a suitable method for achieving this objective. Some parameters which can be examined for process optimization and scale-up are gas holdup, shear stress, fluid velocity, and relations between the process characteristics. Increasing the gas flow rate has a direct relation to the production rate. The flow pattern in a bioreactor is generally dependent on the diameter of the bioreactor and the flow rate of the liquid and gas. Simulation of biochemical operations is not as easy as usual chemical processes; however, it can reduce the number of necessary experiments and speed up the scale-up process. To conclude, the 2 studied types of gas fermenters, i.e., the Vertical tubular Loop Bioreactor (VTLB) and the Horizontal tubular Loop Bioreactor (HTLB) with forced liquid flow, were found to be well-suited reactor configurations. In this review article both experimental and theoretical values from the literature were compared to other reactor types, and aspects of scale-up of gas fermentation were discussed. Gas fermentation of CH_4_ from natural gas, biomass and waste streams to produce P3HB and other PHA is a promising route to make these biopolymers more cost-effective and thereby suitable for more products, replacing conventional fossil plastics. P3HB could be used much more broadly than today when being manufactured in a large-case, cost-effective gas fermentation process from CH_4_. In fact, bioplastics have the technical potential to replace 90% of the fossil polymer volume. Methanotrophic processes and PHA can pave the way in this transition, being biobased and fully biodegradable, hence avoiding microplastics and nanoplastics related issued from the root-cause.
